# Exploring the *In Vitro* Effects of Zingerone on Differentiation and Signalling Pathways in Bone Cell Lines

**DOI:** 10.3390/metabo14120693

**Published:** 2024-12-09

**Authors:** Brunhildé De Vos, Abe E. Kasonga, Anna M. Joubert, Trevor T. Nyakudya

**Affiliations:** Department of Physiology, School of Medicine, Faculty of Health Sciences, University of Pretoria, Private Bag X323, Gezina, Pretoria 0031, South Africa; u21782386@tuks.co.za (B.D.V.); abe.kasonga@up.ac.za (A.E.K.); annie.joubert@up.ac.za (A.M.J.)

**Keywords:** bone metabolism, *in vitro* physiology, osteoblast, osteoclast, zingerone

## Abstract

Objective: Ensuring adequate bone health is crucial for preventing conditions such as osteoporosis and fractures. Zingerone, a phytonutrient isolated from cooked ginger, has gained attention for its potential benefits in bone health. This study evaluated the osteoprotective potential of zingerone and its effects on differentiation and signalling pathways *in vitro* using SAOS-2 osteosarcoma and RAW264.7 macrophage cell lines, aiming to elucidate its mechanism of action in bone remodelling. Methods: SAOS-2 osteosarcoma and RAW264.7 macrophage cells were treated with zingerone at concentrations of 200 µM. Osteoblast differentiation was assessed by alkaline phosphatase (ALP) activity, bone mineralisation via Alizarin Red S stain, and gene expression markers (*ALP*, runt-related transcription factor 2 (*Runx2*), and osteocalcin) via quantitative polymerase chain reaction (q-PCR). Osteoclast differentiation was evaluated by tartrate-resistant acid phosphatase (TRAP) staining, TRAP activity, and mitogen-activated protein kinase (MAPK) pathways. Results: Treatment with zingerone was non-toxic at 200 µM. Zingerone (200 µM) significantly stimulated the gene expression of *ALP* and *Runx2* in SAOS-2 cells (*p* < 0.05) without statistically significantly enhancing SAOS-2 mineralisation via calcium deposits. Moreover, zingerone significantly inhibited osteoclast differentiation in RAW264.7 cells as evidenced by reduced TRAP staining and activity (*p* < 0.05). Conclusions: Zingerone shows promise in reducing osteoclast activity and supporting early osteoblast differentiation, suggesting its potential as a dietary supplement for bone health. *Further in vivo* and clinical studies are needed to confirm its role in managing osteoporosis.

## 1. Introduction

Osteoporosis is a skeletal disorder that is characterised by the progressive degeneration of the bone microarchitecture [1]. The asymptomatic nature of osteoporosis allows it to progress without noticeable symptoms until a fracture occurs, leading to substantial morbidity rates [2]. Estimates indicate that osteoporosis-related fractures will rise significantly in the next two decades, placing a significant burden on healthcare systems [3].

Osteoporosis involves an imbalance in the bone turnover rate where the rate of bone resorption (osteoclasts) exceeds the rate of bone formation (osteoblasts) resulting in a net loss in bone mass [4]. Osteoclasts play a crucial role in bone resorption by secreting acids and enzymes to absorb the bone matrix [5]. Osteoclast differentiation is mainly regulated by receptor activator of nuclear factor kappa-B ligand (RANKL), produced by bone-forming osteoblasts, which binds to the receptor activator of nuclear factor kappa-B (RANK) receptor on the surface of osteoclast precursors [6]. RANKL stimulates key mitogen-activated protein kinases (MAPKs) such as extracellular signal-regulated kinase (ERK), *c*-Jun N-terminal kinase (JNK), and p38 MAPK, and further activates the nuclear factor kappa-B (NF-κB) pathway to promote osteoclast proliferation, differentiation, activation, and cell survival [7]. These pathways result in the expression of nuclear factor of activated T-cells, cytoplasmic 1 (*NFATc1)*, via Fos proto-oncogene (*c-Fos*), which is a major regulator of osteoclastogenesis [8]. *NFATc1* stimulates transcription of osteoclast-specific markers like tartrate-resistant acid phosphatase (TRAP), a biomarker for osteoclast differentiation and bone resorption [8,9]. Compounds that reduce TRAP activity or inhibit specific MAPKs are being investigated as potential treatments for osteoporosis [10].

Runt-related transcription factor 2 (*Runx2*) is the master transcription factor for osteoblasts, essential for activating the translation of genes such as alkaline phosphatase (*ALP*) and osteocalcin (*OC*) that promote osteoblast maturation and function [11]. *Runx2* expression peaks in immature osteoblasts and diminishes in mature osteoblasts, illustrating its pivotal role in determining the lineage and function of osteoblasts [12]. During early osteoblastogenesis, ALP activity is crucial for bone mineralisation by releasing inorganic phosphate ions (Pi’s) that form hydroxyapatite crystals [13]. The mature osteoblast marker (OC) is secreted during the later stages of bone differentiation and mineralisation, and plays a critical role in skeletal development and bone remodelling [14]. The precise coordination of *Runx2*, *OC*, and *ALP* is crucial for osteoblast differentiation and bone formation, providing valuable insights into the efficacy of osteoprotective agents such as zingerone [15].

**Figure 1:**
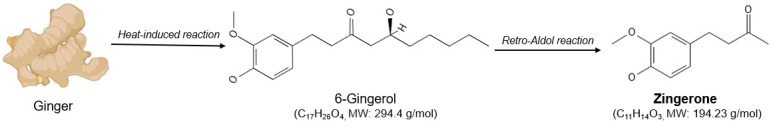
Synthesis of zingerone during ginger processing. In the process, 6-Gingerol is formed by cooking or drying ginger root which then produces zingerone through a retro-aldol reaction. Zingerone is not present in fresh ginger. Chemical structures were sketched with PubChem Sketcher V2.4 (https://pubchem.ncbi.nlm.nih.gov//edit3/index.html accessed on 27 October 2024). MW: molecular weight.

Zingerone (4-(4-hydroxy-3-methoxyphenyl)-2-butanone), a compound found in cooked ginger (Figure 1), is renowned for its antioxidant, anti-inflammatory, anti-cancer, and hypoglycaemic properties [16,17]. These attributes are particularly significant for bone health, as oxidative stress and chronic inflammation are major contributors to bone loss and the pathogenesis of conditions such as osteoporosis [18]. The increase in reactive oxygen species (ROS) and inflammation disrupts the balance of bone remodelling by impairing osteoblast differentiation and promoting RANKL-induced osteoclastogenesis, leading to excessive bone resorption [18]. Through its anti-inflammatory effects, zingerone may have the potential to preserve bone integrity by protecting osteoblast function, reducing osteoclastogenesis and maintaining bone homeostasis [19].

Recent studies have highlighted zingerone’s potential *in vitro* benefits for bone health [20,21,22]. For instance, zingerone has been shown to suppress osteoclast formation in RAW264.7 cells by downregulating the NF-κB signalling pathway, potentially preventing ROS-induced bone resorption [21]. The activation of RANKL exacerbates the production of ROS, inhibiting osteoblastic differentiation markers of *ALP*, *OC*, and *Runx2* [18]. Additionally, Shamsabadi et al. (2023) indicated that the anti-inflammatory properties of zingerone may have provided additional protective effects against bone loss by inhibiting the production of proinflammatory mediators such as interleukin (IL), IL-6, IL-1β, and tumour necrosis factor alpha (TNF-α), in turn attenuating oxidative stress and preventing apoptosis [23,24]. Furthermore, Song et al. (2020) found that zingerone promotes osteoblast differentiation through miR-200c-3p upregulation and smad7 downregulation in human bone marrow mesenchymal stem cells (hBMSC) [20]. This regulation enhances the expression of osteoblast differentiation markers such as *ALP*, *Runx2*, and *OC* [20]. Both oxidative stress and inflammation negatively affect bone homeostasis leading to the development of osteoporosis [18]. The combined bioactive effects of zingerone may synergistically benefit bone health by inhibiting RANKL-induced ROS production via MAPK and NF-κB signalling pathways [23,25].

Given these health benefits, further *in vitro* research is warranted to explore zingerone’s molecular interactions in bone metabolism and its osteogenic potentials. This study investigated the *in vitro* osteogenic potential of zingerone using SAOS-2 osteosarcoma and RAW264.7 macrophage cell lines.

## 2. Materials and Methods

### 2.1. Zingerone Sample Preparation

Stock solutions of 0.1 M zingerone (Alfa Aeasar; Haverhill, MA, USA) were prepared in dimethyl sulfoxide (DMSO) and stored at −80 °C. The stock solution was diluted to concentrations ranging from 0.1–200 µM in McCoy’s 5A or Dulbecco’s Modified Eagle Medium (DMEM) media (GIBCO; Carlsbad, CA, USA) [20,22].

### 2.2. Cell Lines, Culture, and Treatment

#### 2.2.1. SAOS-2 Cells

The human osteosarcoma-derived cell line, SAOS-2 (European Collection of Cell Culture^®^, Sigma-Aldrich, CAT# 89050205, St. Louis, MO, USA), was used as a human osteoblast-like cell line [26]. This was due to its osteoblastic characteristics and ability to form mineral deposits and express several key markers of osteoblast differentiation [27,28]. It was used as an *in vitro* model for bone-related research to explore osteoblast activity and differentiation [29]. SAOS-2 cells were cultured and maintained in complete McCoy’s 5A containing 20% heat-inactivated foetal bovine serum (FBS) (Capricorn Scientific; Ebsdorfergrund, Giessen, Hessen, Germany) at 37 °C in 5% CO_2_ humidified atmosphere. Cells with a confluency of 70–80% were trypsinised with 0.25% trypsin–ethylenediaminetetraacetic acid (EDTA), centrifuged for 2 min at 1000× *g*, and resuspended in McCoy’s 5A [30]. Following trypan blue exclusion method, cells were then seeded for subsequent experiments [31].

#### 2.2.2. RAW264.7 Cells

RAW264.7 murine macrophages (American Type Culture Collection; Rockville, MD, USA) were used as an osteoclast precursor cell line [32]. These cells express the RANK receptor and can form large, multinucleated, bone-resorbing cells in the presence of 5 ng/mL RANKL (R&D Systems, CAT# 472-TEC-010, Minneapolis, MN, USA) [33,34]. RAW264.7 cells were cultured in complete DMEM supplemented with 10% heat-inactivated FBS and incubated at 37 °C and 5% CO_2_. For experimental seeding, cells were detached using a cell scraper and seeded for subsequent experiments following trypan blue exclusion [30].

### 2.3. Cell Viability and Proliferation: Resazurin Assay

The effect of zingerone on bone cell viability was evaluated via resazurin assay. SAOS-2 or RAW264.7 cells were seeded separately in a 96-well plate containing complete McCoy’s 5A and DMEM media at a density of 2.5×10^4^ cells/mL overnight. Subsequently, media were replaced, and the cells were exposed to zingerone (0.1–200 µM) or the vehicle (0.1% DMSO) for 48 h. The positive control was replicated by exposing cells to 0.2% triton X-100. Media were replaced with 10% resazurin solution and incubated for 4 h at 37 °C [35]. Absorbance was measured at 570 nm and 600 nm using an Epoch microplate spectrophotometer (BioTek Instruments Inc., Winooski, VT, USA) [36].

### 2.4. Alizarin Red S Staining and Alkaline Phosphatase (ALP) Activity Assay

SAOS-2 cells were seeded in sterile 96-well plates at a density of 2.5×10^4^ cell/mL in osteogenic differentiation medium (osMcCoy) (McCoy’s 5A medium supplemented with 10 mM β-glycerophosphate, 50 mM ascorbic acid, and 10 mM dexamethasone) and different concentrations of zingerone (5, 100, and 200 µM) or the vehicle (0.1% DMSO) for 7, 14, and 21 days. These time points were used as correspondents to the three critical stages of the differentiation process. Therefore, the effects of zingerone on SAOS-2 cells were evaluated on early- (day 7), intermediate- (day 14), and late-stage (day 21) mineralisation. Media and all factors were changed every 2–3 days.

Cells were fixed with 10% formalin for 1 h and stained with 2% (*w*/*v*) Alizarin Red S (Merck, Darmstadt, Germany) for 10 min [37]. Cells were then rinsed with double-distilled water and air-dried overnight before images were captured using an Olympus BH2 microscope equipped with an SC30 camera (Olympus Corporation; Hachioji-shi, Tokyo, Japan). Alizarin Red S dye was extracted with 10% acetic acid, neutralised with 10% ammonium hydroxide, and absorbance was measured at 405 nm using an Epoch microplate spectrophotometer (BioTek Instruments Inc.; Winooski, VT, USA). Cells were fixed for 5 min with 10% formalin following 1 h incubation with ALP buffer (5 mM *p*-nitrophenylphosphate, 0.5 mM MgCl_2_·6H_2_O, 0.1% triton-X 100 in 50 mM Tris-HCl at pH 9.5) at 37 °C. Thereafter, 100 µL of the product solution was transferred into a sterile 96-well plate and absorbance was measured at 405 nm and 650 nm using an Epoch microplate spectrophotometer (BioTek Instruments Inc.; Winooski, VT, USA). Data were calculated relative to the vehicle.

### 2.5. Quantitative Polymerase Chain Reaction (q-PCR)

SAOS-2 cells were seeded in 48-well plates at a density of 3 × 10^4^ cells/mL with osMcCoy containing zingerone (200 µM) or the vehicle (0.1% DMSO) for 7 and 14 days. The sub-maximal effective concentration of zingerone was selected based on the proliferation and differentiation assays. The expression of mRNA was evaluated at two different time points to capture the shifts in gene expression that characterise different stages of osteoblast differentiation. Day 7 represents an early- to mid-stage in osteoblast differentiation where the cells are beginning to express early markers of osteogenesis such as *Runx2* and *ALP* (alkaline phosphatase), whilst day 14 is where SAOS-2 cells typically exhibit more mature osteoblast characteristics, including increased expression of late-stage markers like osteocalcin (*OC*), as an indication of the progression towards mineralisation. Total ribonucleic acid (RNA) was extracted using an Aurum^TM^ Total RNA Mini Kit (BIO-RAD Laboratories, Hercules, CA, USA) according to the manufacturer’s instructions. RNA was reverse-transcribed to cDNA using a SensiFAST cDNA Synthesis Kit (Bioline Reagents Ltd., London, UK). The osteoblast-specific primers (Table 1) were sourced from Inqaba Biotec (Pretoria, South Africa). Quantitative PCR was performed via Luna^®^ Universal q-PCR Master Mix (New England Biolabs, Ipswich, MA, USA) and Light Cycler Nano (Roche Diagnostics; Basel, Switzerland) according to the protocol indicated in Table 2. Data were analysed using the −2^ΔΔCT^ method with Ribosomal Protein Lateral Stalk Subunit P0 (*RPLP0*) as the loading control.

### 2.6. Tartrate-Resistant Acid Phosphatase (TRAP) Staining and Activity

RAW264.7 osteoclast-like cells were seeded in sterile 96-well culture plates at 2.5 × 10^4^ cells/mL in complete DMEM together with 5 ng/mL RANKL. A lower concentration of RANKL was used to maintain cell viability and ensure that the observed effects were due to differentiation and not cell loss. This allowed for a clear and measurable readout of TRAP activity in response to zingerone treatment that directly correlated with the number and activity of mature osteoclast-like cells. RAW264.7 cells were exposed to zingerone (100, 150, and 200 µM) or the vehicle (0.1% DMSO) for 5 days. Media and all factors were replaced on days 2–3. Thereafter, cells were fixed with 3.7% formaldehyde in PBS and stained for TRAP as described by Kasonga et al. (2015) Images were captured (scale bar = 2 mm) with an Olympus BH2 microscope (Olympus Corporation, Tokyo, Japan) and TRAP-positive cells with three or more nuclei were counted as osteoclasts [38]. Furthermore, conditioned media were collected from each well and TRAP enzyme activity was measured at 405 nm and 650 nm using an Epoch microplate spectrophotometer (BioTek Instruments Inc.; Winooski, VT, USA) [5].

### 2.7. Western Blotting

RAW264.7 cells were seeded in 6-well plates at 5 × 10^4^ cells/mL containing complete DMEM and incubated overnight to attach. Cells were pre-exposed to the sub-maximal effective concentration of zingerone (200 µM) or the vehicle (0.1% DMSO) for 4 h at 37 °C. For western blot analysis, a higher RANKL concentration (15 ng/mL) was used to ensure that the specific proteins associated with osteoclast differentiation and activation were sufficiently expressed. This concentration produced a clear and detectable signal that ensured a strong activation of the investigated pathways and proteins therefore mitigating low protein expression with weak or undetectable bands on the membrane. After stimulation with RANKL (15 ng/mL) for 5, 10, and 15 min, cells were lysed in ice-cold radio immunoprecipitation assay (RIPA) buffer supplemented with 0.3 M phenylmethyl-sulfonyl fluoride (PMSF), 5% protease inhibitor cocktail, and 5% phosphatase inhibitor cocktail. Purified proteins were quantified using a micro bicinchoninic acid (BCA) protein kit (Pierce Biotechnology, Rockford, IL, USA), fixed on a 4–15% Mini Protean TGX gel (Bio RAD, Hercules, CA, USA) at 200 mV, and electro-transferred to nitrocellulose membranes using Tris-glycine transfer buffer (25 mM Tris, 192 mM glycine and 20% methanol) for 1 h. Membranes were blocked with 5% bovine serum albumin for 1 h and probed with primary rabbit polyclonal antibodies (1:500; *p*-p38, *p*-JNK, *p*-ERK, and 1:1000; JNK, ERK, and p38) (Abcam, Waltham, MA, USA) overnight at 4 °C. Glyceraldehyde-3-phosphate dehydrogenase (GAPDH) served as the loading control. After incubation with goat anti-rabbit HRP conjugates secondary antibody (Bio RAD, Hercules, CA, USA) for 1 h at room temperature, membranes were developed using Clarity ECL substrate and visualised via Chemidoc MP (Bio RAD, Hercules, CA, USA). Band densities were quantified using ImageJ software (NIH, Version 1.53r) [39].

### 2.8. Statistical Analysis

All data were displayed as mean ± standard deviation (SD) unless stated otherwise. All experiments were conducted in triplicate. Statistical analysis was performed with the aid of GraphPad Prism 9 software (San Diego, CA, USA). Differences were analysed using two-way ANOVA following Tukey’s multiple comparison. Data were considered statistically significant if *p* < 0.05.

## 3. Results

### 3.1. Zingerone Does Not Affect Mineralisation or ALP Protein Activity in SAOS-2 Cells

**Figure 2:**
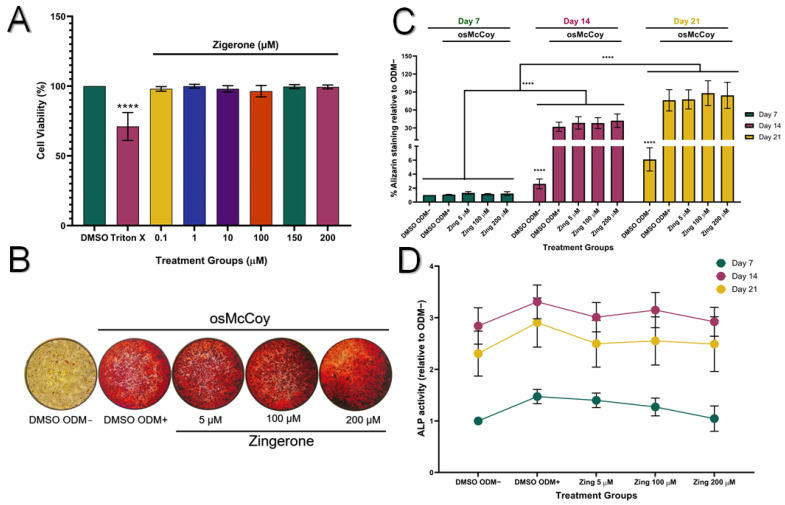
Effect of zingerone on cell viability, mineralisation, and ALP activity in SAOS-2 cells. (**A**) Zingerone (0.1–200 µM) effects on cell viability (%) of undifferentiated SAOS-2 cells following 48 h treatment. Triton X-100 (0.2%) served as the positive control for cytotoxicity. (**B**) Mineralisation of SAOS-2 cells treated with zingerone (5–200 µM) for 7–21 days, evaluated using Alizarin Red S staining and measured at 540 nm. (**C**) SAOS-2 cells treated with osMcCoy media with or without zingerone for 21 days. (**D**) Zingerone’s effect on ALP activity as a marker of osteoblast-like differentiation measured over three stages of differentiation: 7, 14, and 21 days. Resazurin assay was used to evaluate cell viability. Data presented as mean ± SD (n = 3) (**A**) or mean ± SEM (**C**,**D**) containing at least three independent experiments. **** *p* < 0.001 (vs. DMSO). Zing: zingerone; DMSO ODM–: undifferentiated media; DMSO ODM+: osteogenic media.

Zingerone showed no cytotoxic effects to SAOS-2 cells at all tested concentrations (Figure 2A; *p* > 0.05). Cells treated with osMcCoy media (ODM+) induced mineralisation and stained pink for Alizarin Red S compared to those grown without osMcCoy (ODM–) (Figure 2B). The influence of zingerone on SAOS-2 differentiation was investigated during three critical stages: 7, 14, and 21 days. Mineralisation of SAOS-2 cells was at its lowest capacity on day 7 and peaked on day 21 (Figure 2C). A significant increase in mineralisation was observed in SAOS-2 ODM+ cells on days 14 and 21 when compared to their corresponding SAOS-2 ODM– controls (*p* < 0.001; Figure 2C). Zingerone (5, 100, and 200 µM) had no effect on promoting osteoblast-like mineralisation of SAOS-2 cells from day 7–21 when compared to the ODM+ cells (*p* > 0.05; Figure 2C).

ALP activity increased significantly on day 14 for all treatment groups compared to day 7 (*p* < 0.05; Figure 2D). However, the ODM– cells showed similar ALP levels as the ODM+ cells on days 7, 14, and 21, indicating that the ALP assay may lack sensitivity in detecting osteoblast-like differentiation of SAOS-2 cells. Zingerone (5–200 µM) did not significantly affect ALP activity at days 7, 14, or 21 when compared to the ODM+ control (*p* > 0.05; Figure 2D).

### 3.2. Zingerone Stimulates Runx2 and ALP Expression

**Figure 3:**
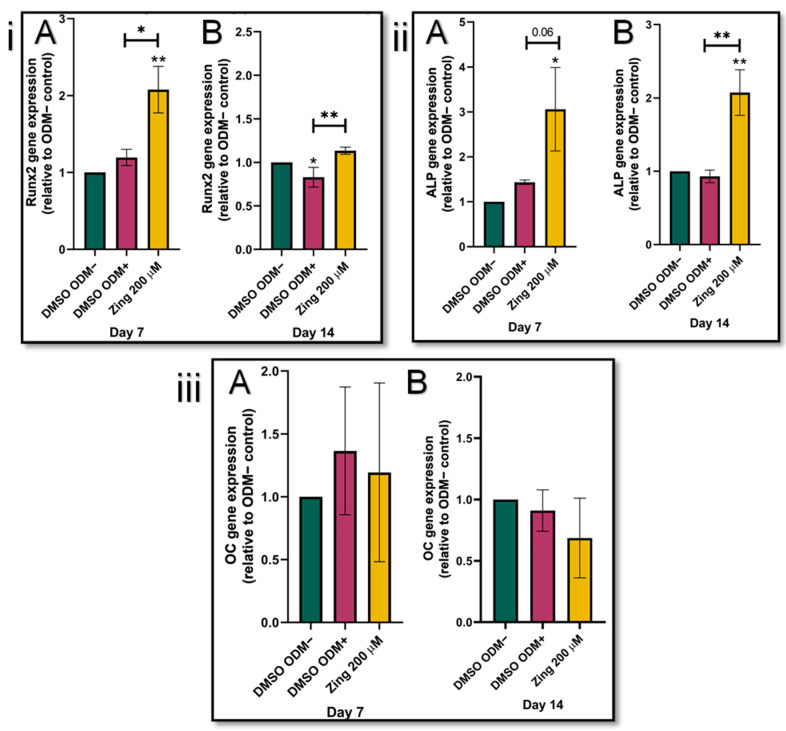
The gene expression of *ALP* (**i**), *Runx2* (**ii**), and *OC* (**iii**) in SAOS-2 cells treated with 200 µM zingerone for 7 and 14 days of differentiation was assessed by q-PCR. Evaluation of the effects of zingerone on the expression of genes involved in the early and intermediate stages of osteoblast differentiation was conducted by exposing cells to osteogenic media for 7 days (**A**) and 14 days (**B**). Data presented as mean ± SD (n = 3), normalised to DMSO ODM–. *^/^** *p* < 0.05 compared to control (DMSO ODM–). Zing: zingerone; DMSO ODM–: undifferentiated media; DMSO ODM+: osteogenic media.

The addition of zingerone (200 µM) significantly increased *Runx2* expression levels in SAOS-2 cells on day 7 and 14 compared to their corresponding ODM+ controls (*p* < 0.05; Figure 3i(A,B). In the early stages of differentiation as represented by day 7 (Figure 3ii(A)), ALP levels of zingerone-treated SAOS-2 cells were two-fold higher (3.06) compared to the osteogenic control without zingerone (ODM+; 1.44) (*p* = 0.06). On day 14, the zingerone-treated SAOS-2 cells had ALP levels significantly higher (2.07) than the (ODM+)-treated control (0.93) (*p* < 0.05; Figure 3i(B)). The *Runx2* and *ALP* gene expression of ODM+ cells were not significantly altered compared to their corresponding ODM– controls (*p* > 0.05; Figure 3i,ii); zingerone had no effect on *OC* gene expression in SAOS-2 cells during the 7- and 14-day differentiation and maturation process (*p* > 0.05; Figure 3iii).

### 3.3. Zingerone Inhibits Osteoclast Formation and Activity in RAW264.7 Macrophages

**Figure 4:**
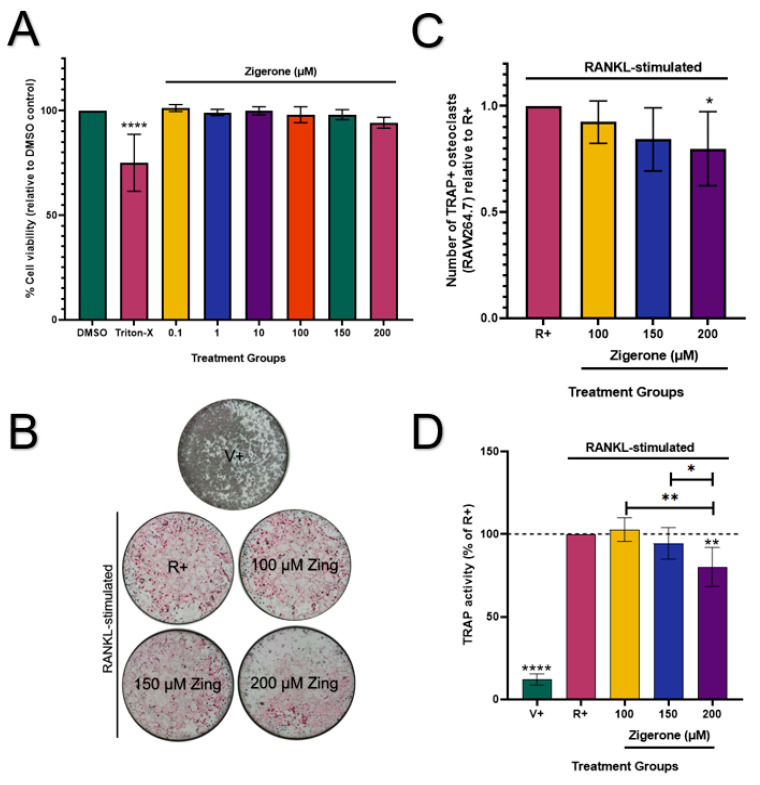
Effect of zingerone on cell viability and osteoclast differentiation in RAW264.7 cells. (**A**) Zingerone’s (0.1–200 µM) effects on cell viability (%) were evaluated by resazurin assay in undifferentiated RAW264.7 cells after 48 h treatment. Triton X-100 (0.2%) served as the positive control for cytotoxicity. (**B**) Microscopic images of TRAP-stained RAW264.7 cells (scale bar = 2 mm) treated with zingerone (100–200 µM) and RANKL (5 ng/mL). (**C**) Quantification of osteoclasts, identified as large multinucleated cells with three or more nuclei, stained pink. (**D**) TRAP activity measured in conditioned media via *p*-NPP substrate and displayed relative to the R+ cells. Data presented as mean ± SD (n = 3). (**A**): **** *p* < 0.05 vs. DMSO. (**C**): * *p* < 0.05 vs. R+. (**D**): **** *p* < 0.05 V+ vs. R+. ** *p* < 0.05 200 µM Zing vs. R+. V+: vehicle control (no RANKL added); R+: RANKL-stimulated cells; Zing: zingerone.

Zingerone (0.1–200 µM) had no cytotoxic effects on RAW264.7 osteoclast-like cells (*p* > 0.05; Figure 4A) when compared to the vehicle. Therefore, these concentrations were used for downstream experiments. Cells that were not treated with RANKL showed no osteoclast formation (Figure 4B). The number of osteoclasts decreased significantly (20%) in cells treated with 200 µM zingerone when compared to the RANKL-positive cells (R+) (*p* < 0.05; Figure 4C). Furthermore, TRAP activity was significantly reduced in the absence of RANKL (V+) compared to all treatments (*p* < 0.0001; Figure 4D). Consistent with the TRAP staining results (Figure 4C), treatment with zingerone (200 µM) resulted in a significant 19.8% decrease in TRAP activity compared to the positive vehicle R+ (*p* < 0.05; Figure 4D).

### 3.4. Zingerone Did Not Significantly Activate the Expression of MAPK Proteins 

**Figure 5:**
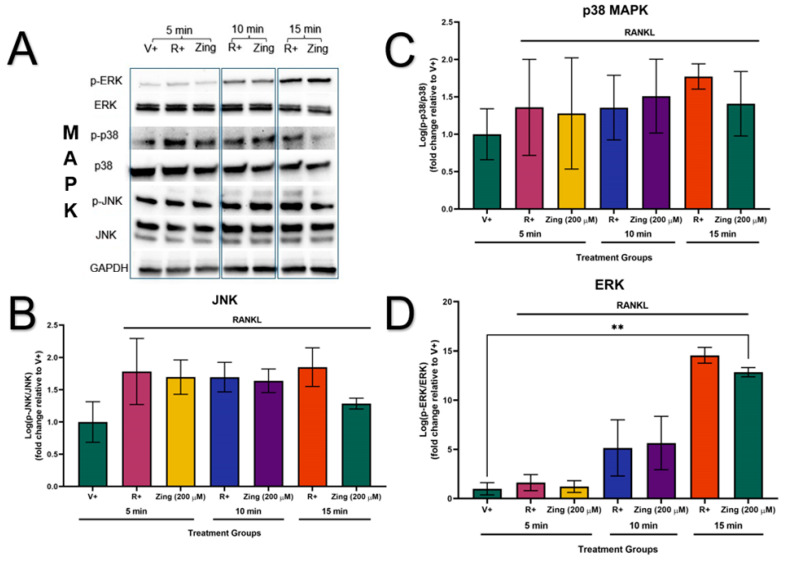
The effects of zingerone (200 µM) on osteoclast-specific protein expression via RANKL-stimulated (15 ng/mL) MAPK (JNK, p38, ERK) signalling pathway. (**A**) Evaluating the effects of 200 µM zingerone on RANKL-stimulated MAPK signalling as represented by membrane images. GAPDH was used as the control (**B**–**D**) The expression levels of MAPKs (JNK, p38, and ERK) in RAW264.7s were treated with 200 µM zingerone and quantified by western blot via cytoplasmic extraction protocol. Data presented as mean ± SD of 3 independent repetitions (n = 3). ** *p* < 0.05 compared to vehicle control (V+). RANKL: receptor activator of nuclear factor kappa beta; JNK: Jun N-terminal kinase; ERK: extracellular signal-regulated kinase; MAPKs: mitogen-activated protein kinases; GAPDH: glyceraldehyde-3-phosphate dehydrogenase; V+: vehicle control (RANKL not present); R+: RANKL-only stimulated control; Zing: zingerone.

The phosphorylation of the MAPK proteins (JNK, p38, and ERK) were not activated in the absence of RANKL stimulation (V+) (Figure 5A). The sub-maximal effective concentration of zingerone (200 µM) did not decrease the expression of *p*-JNK/JNK, *p*-p38/p38, and *p*-ERK/ERK (*p* > 0.05; Figure 5B–D). However, the membranes showed that zingerone could potentially supress the activation of JNK, p38, and ERK at 15 min of RANKL stimulation compared to their corresponding R+ controls at 15 min (Figure 5A).

## 4. Discussion

Osteoporosis is a progressive bone degenerative disorder characterised by overactive osteoclasts and underactive osteoblasts. Although drug therapies are available, these are often costly and not readily available to individuals in low-income countries. This study investigated the *in vitro* molecular mechanisms of zingerone on both RAW264.7 and SAOS-2 bone cell lines to assess its potential osteoprotective effects in bone health. These cells are routinely used as *in vitro* models to study the potential osteoprotective properties that are crucial for evaluating therapeutic agents in bone [33]. Zingerone, a bioactive compound constituting approximately 9% of cooked ginger, is not present in fresh ginger but is formed through the thermal degradation of 6-gingerol which occurs during the drying or cooking process of ginger [40]. Zingerone is known for its antioxidant and anti-inflammatory properties, suggesting potential benefits for bone health [41,42]. *In vitro* studies, such as this present study, play a crucial role in advancing our understanding of cellular processes within a controlled experimental environment. Through the systematic testing of zingerone under varying experimental conditions, we aim to deepen our comprehension of its mechanisms of action on both osteoblasts and osteoclasts, providing valuable insights into its potential biological effects.

The formation and mineralisation of bone tissue depend on the vital production of ALP, predominantly expressed in osteoblastic cells as a marker of osteoblast differentiation [43]. To investigate zingerone’s effect on osteoblast function, markers of mineralisation (Alizarin Red S stain) and ALP activity were determined after 7, 14, and 21 days. Findings from this study showed that zingerone (5–200 µM) did not significantly enhance SAOS-2 mineralisation, as evident by calcium deposits, or significantly increase ALP activity in comparison to control group. Nonetheless, our study did indicate a slight increase in mineralisation with zingerone concentrations of 100 and 200 µM from day 7 to 21; however, this was not statistically significant. The precise mechanisms influencing ALP activity in SAOS-2 cells were not examined in this study, as one limitation, and warrant further investigation. Our study did show that 200 µM of zingerone significantly upregulated *ALP* gene expression on days 7 and 14. Given the inherently high levels of ALP protein in SAOS-2 cells, the observed increase in *ALP* gene expression did not correspond to higher ALP activity, possibly due to assay saturation or a potential temporal delay between changes in gene expression and subsequent enzyme activity [29,44]. The observed inverse relationship between ALP activity and *ALP* messenger RNA (mRNA) has been previously reported by Kyeyune-Nyombi et al. (1995), suggesting that zingerone could potentially advance bone health by influencing bone mineralisation at a genetic level, without immediately increasing ALP protein activity [44]. The complexity of the relationship between gene expression and enzyme activity suggests that the osteoprotective effects of zingerone may take longer to manifest or require different conditions, such as other cell lines with lower baseline ALP activity. Therefore, our data indicated that the effects of zingerone to regulate ALP activity in SAOS-2 cells did not parallel changes in *ALP* gene expression. These mechanistic insights may be crucial for future investigations, especially related to osteoporosis research. In addition, our study indicated that the treatment of SAOS-2 cells with zingerone (200 µM) significantly upregulated *Runx2* expression on days 7 and 14, a key transcription factor involved in early osteoblast differentiation and mineralisation [22]. *Runx2* plays a crucial role in regulating osteoblast-specific gene expression such as *ALP* and *OC*, facilitating matrix mineralisation and guiding osteoblast maturation [45]. The upregulation of both *ALP* and *Runx2* suggests that zingerone has potential beneficial effects on promoting factors that are essential for osteoblast differentiation and maturation [18]. Despite the observed increase in *Runx2* gene expression, zingerone did not significantly enhance mineralisation or affect gene expression of *OC*, another osteogenic marker linked to late-stage osteoblastogenesis [46]. The lack of effect on *OC* expression may potentially suggest that zingerone regulates early stages of osteoblast differentiation in SAOS-2 cells, without directly influencing later stages of differentiation. Future studies should investigate the specific role of zingerone during the final stages of matrix maturation and mineralisation by extending the differentiation period to 21 days and including osteoblastic marker genes involved in late-stage differentiation, with particular emphasis on the regulation of *OC,* type 1 collagen (*Col1a1*), bone sialoprotein (*BSP*), and osteopontin (*OPN*) to further elucidate its underlying mechanisms on osteoblastogenesis [14,47].

The mechanism of action of zingerone was further evaluated in an osteoclast-like cell model using RAW264.7 murine macrophages [48]. Osteoclast differentiation was induced by 5 ng/mL of RANKL, while a higher concentration of 15 ng/mL RANKL was used for western blot analyses to ensure robust protein expression, enabling clear detection of key signalling molecules and differentiation markers. The difference in RANKL concentrations reflects the distinct objectives and sensitivity requirements of each experimental approach. Decreased concentrations of RANKL allowed for a more nuanced and controlled study of zingerone’s effects on osteoclast differentiation in RAW264.7 cells. Previous studies have successfully induced osteoclastogenesis in RAW264.7 cells with RANKL concentrations ranging between 0.5 to 100 ng/mL [49,50]. In the current study, zingerone (200 µM) effectively inhibited the activity of TRAP and significantly reduced the number of TRAP-positive osteoclasts in RAW264.7 cells. The ability of zingerone to suppress osteoclast function suggests potential benefits for conditions involving excessive bone resorption such as osteoporosis. Similar reports have shown that zingerone, at much higher concentrations (150–600 µM), was able to significantly inhibit TRAP activity and attenuate the expression of MAPK proteins in RAW264.7 cells [21]. These findings stand in contrast to our results, where zingerone demonstrated cytotoxic effects on RAW264.7 cells at concentrations exceeding 200 µM. Furthermore, no significant differences were observed in the expression levels of *p*-JNK/JNK, *p*-p38/p38, and *p*-ERK/ERK with or without zingerone (200 µM) treatment. The inhibition effect observed by Yang et al. (2023) might be attributed to laboratory-specific environmental factors unique to their laboratory and not necessarily to the ability of zingerone to inhibit osteoclastogenesis [21]. Some of the observed divergences may have been caused by genetic and epigenetic cellular diversity, including variability in the source and purity of zingerone itself [21]. The differences in the composition of the cell culture medium may have been the distinguishing factor. Previous studies have shown that media supplementation with antibiotics such as penicillin-streptomycin (PenStrep) can significantly alter gene expression and transcription factors [51,52]. In the present study, these potential metabolic alterations were avoided by excluding antibiotics from our culture medium, whereas Yang et al. (2023) not only used a different culture medium but also supplemented alpha-Minimum Essential Medium Eagle (MEM) with 1% PenStrep, which may have affected the expression of MAPK signalling pathways [21,51,52].

The effects of zingerone on both bone cell lines, SAOS-2 and RAW264.7, are multifaceted and involve a combination of different mechanisms associated with its antioxidant and anti-inflammatory potencies [20,22,53]. This *in vitro* study suggests that zingerone, similar to other osteoclast-inhibiting agents (e.g., zoledronate), may target only one specific osteoclastogenic mechanism such as TRAP activity without significantly influencing other osteoclastogenic pathways [54]. Future studies may consider incorporating metabolites such as short-chain fatty acids (SCFAs), polyamines, and hydrogen sulfide (H_2_S), which play critical roles in the metabolism of osteoblasts and osteoclasts, as controls [55,56]. Including these metabolites could provide a more robust validation of zingerone and offer deeper insights into its mechanism of action as a potential anti-osteoclastic agent. This present study also demonstrated how zingerone contributed to the early stages of osteoblast differentiation by promoting the expression of *ALP* and *Runx2* in SAOS-2s. Addressing these specific areas contributes to the growing body of knowledge on the molecular mechanism of zingerone and its potential role in bone cells, while also offering novel perspectives and experimental insights that could inspire further research in the field. The overall efficacy of zingerone as a bone health supplement may depend on its bioavailability, concentration, duration of exposure, and interaction with other factors that affect bone metabolism such as culture medium, cell line, and cell density [52]. The hydrophobic nature of zingerone has been shown to limit its pharmacological effects *in vitro*, emphasising the need for future research to explore nano-formulations or bioenhancers with zingerone to improve its bioavailability in cell models [57]. This study provides a foundational understanding of zingerone’s cellular effects on osteoblast and osteoclast differentiation. However, future research should focus on providing more in-depth mechanistic insight of its effects on signalling pathways such as RANKL/OPG and Wnt/β-catenin. In addition, future research needs to evaluate the osteogenic properties of zingerone *in vivo*, by quantifying bone density and structural integrity of zingerone-treated bones using advanced non-destructive micro-computed tomography (micro-CT) techniques, to allow precise assessments of parameters such as trabecular thickness, porosity, and cortical bone integrity [58].

## 5. Conclusions

We have shown that zingerone exhibits moderate *in vitro* potentials as an osteogenic compound. At a concentration of 200 µM, zingerone was able to reduce RANKL-induced osteoclastogenesis in RAW264.7 cells through TRAP inhibition whilst also enhancing early stages of osteoblast differentiation by upregulating the gene expression of *ALP* and *Runx2* in SAOS-2s. However, its impact on MAPK signalling in RAW264.7 cells and later stages of osteoblast differentiation in SAOS-2 cells were less potent. Our findings suggest an alternative hypothesis regarding how zingerone affects bone metabolism, particularly in relation to osteoclastogenesis. The moderate anti-osteoclastic effects of zingerone suggest that it may potentially aid in preserving bone mass by reducing bone resorption. While prior research has focused on some of zingerone’s osteoclast-inhibiting properties, we explore whether its osteogenic potentials could extend to osteoblastic cell lines. The mechanism of action exhibited by zingerone in the current study provides new insight into how zingerone modulates bone cell activity. These findings highlight the potential of zingerone as a dietary supplement for maintaining bone health. Future research should incorporate gut-derived metabolites as controls to strengthen the validation of zingerone and elucidate its molecular mechanisms in bone metabolism. Additionally, future *in vivo* and clinical research may be necessary to elucidate the clinical relevance of zingerone as a supplement for bone health.

## Figures and Tables

**Table 1:** Forward and reversed primers used for q-PCR. Investigating the effect of zingerone on gene expression of *Runx2*, *ALP*, and *OC* during two stages of SAOS-2 differentiation: early (day 7) and late (day 14).

Gene	Forward Primer Sequence (5′—3′)	Reverse Primer Sequence (5′—3′)
*RPLP0* (housekeeping)	GAAACTGTTTAACTTCGCTTCC	GACTCGTTTGTACCCGTTGATG
*Runx2*	GCTGTTATGAAAAACCAAGT	GGGAGGATTTGTGAAGAC
*ALP*	ACGTGGCTAAGAATGTCATC	CTGGTAGGCGATGTCCTTA
*OC*	ATGAGAGCCCTCACACTCCTC	CCGTAGAAGCGCCGATAGGC

**Table 2:** Cycles, temperature, and duration used for q-PCR protocol using the Light Cycler Nano.

Cycles	Temperature	Duration	Step
1	95 °C	1 min	Initial denaturing
45	95 °C	15 s	Denaturing
	60 °C	30 s	Annealing
	72 °C	15 s	Extension
1	72 °C	1 min	Melting curve

## Data Availability

The original contributions presented in the study are included in the article, further inquiries can be directed to the corresponding author upon reasonable request.
